# Nanotechnology as a new strategy for the diagnosis and treatment of gliomas

**DOI:** 10.7150/jca.96859

**Published:** 2024-07-02

**Authors:** Jun Lei, Yiyang Huang, Yichuan Zhao, Zhi Zhou, Lei Mao, Yanhui Liu

**Affiliations:** 1 Department of Neurosurgery, The First People's Hospital of Shuangliu District (West China Airport Hospital of Sichuan University), Chengdu 610200, China.; 2 Department of Neurosurgery, West China Hospital of Sichuan University, Chengdu 610041, China.; 3 Southwest Medical University, Luzhou 646000, China.

**Keywords:** glioma, glioblastoma, blood‒brain barrier, nanotechnology, nanoparticles

## Abstract

Glioma is the most common malignant tumor of the central nervous system (CNS), and is characterized by high aggressiveness and a high recurrence rate. Currently, the main treatments for gliomas include surgical resection, temozolomide chemotherapy and radiotherapy. However, the prognosis of glioma patients after active standardized treatment is still poor, especially for glioblastoma (GBM); the median survival is still only 14.6 months, and the 5-year survival rate is only 4-5%. The current challenges in glioma treatment include difficulty in complete surgical resection, poor blood‒brain barrier (BBB) drug permeability, therapeutic resistance, and difficulty in tumor-specific targeting. In recent years, the rapid development of nanotechnology has provided new directions for diagnosing and treating gliomas. Nanoparticles (NPs) are characterized by excellent surface tunability, precise targeting, excellent biocompatibility, and high safety. In addition, NPs can be used for gene therapy, photodynamic therapy, and antiangiogenic therapy and can be combined with biomaterials for thermotherapy. In recent decades, breakthroughs in diagnosing and treating gliomas have been made with various functional NPs, and NPs are expected to become a new strategy for glioma diagnosis and treatment. In this paper, we review the main obstacles in the treatment of glioma and discuss the potential and challenges of the latest nanotechnology in the diagnosis and treatment of glioma.

## Introduction

The annual prevalence of gliomas is 3-6.4 / 100,000 people, accounting for 23.3% of all central nervous system (CNS) tumors and 78.3% of malignant tumors. The World Health Organization (WHO) classifies gliomas into grades I-IV according to the degree of malignancy 1. Of these, glioblastoma (GBM) has the worst prognosis, with a median overall survival (OS) of approximately 15 months 2,3. The standard treatment options for glioma include surgical resection and temozolomide (TMZ) radiotherapy. However, even with this combination, patients with glioma still have a poor prognosis and a high recurrence rate 4. The invasive growth of gliomas makes complete surgical resection a challenge, which is the main reason for the postoperative recurrence of gliomas and the necessity of adjuvant therapy 4,5. On the other hand, the blood-brain barrier (BBB), therapeutic resistance, and genetic heterogeneity are the main challenges currently facing glioma treatment 6-9. Therefore, the development of new approaches for the treatment and diagnosis of gliomas is urgent and necessary.

Nanotechnology refers to the study of the properties and interactions of substances at the atomic and molecular levels at the nanoscale (1-100 nanometers), and the use of these properties to intersect with multiple disciplines 10. With the continuous development of nanotechnology in recent decades, nanotechnology has shown great promise in diagnosing and treating glioma. Nanoparticles (NPs) have shown potential advantages in a variety of cancer therapies due to various benefits such as their small size, modifiable surface, and few toxic side effects 11,12. NPs with the appropriate modifications can successfully deliver drugs to the brain, which is the most important advantage of NPs for CNS disease treatment. For example, lipid-based NPs (LBNPs) with low immunogenicity, high biocompatibility, and enhanced BBB crossing ability have become the main nanocarriers for intracranial disease drug delivery 13. In addition, nanocarrier systems for delivering glioma-specific receptor inhibitors can efficiently inhibit the signaling axis abnormally activated by gliomas, thereby reducing glioma therapeutic resistance and preventing its progression 14. Some magnetic NPs (MNPs) have uniquely high permeability and distinctive and prominent magnetism when subjected to magnetic fields have been used for detection of biomarkers, and may become an important means for the diagnosis of a variety of diseases, including glioma 15-18. In short, NPs have a wide range of applications and are expected to become a new modality for glioma diagnosis and treatment.

**Figure 1:**
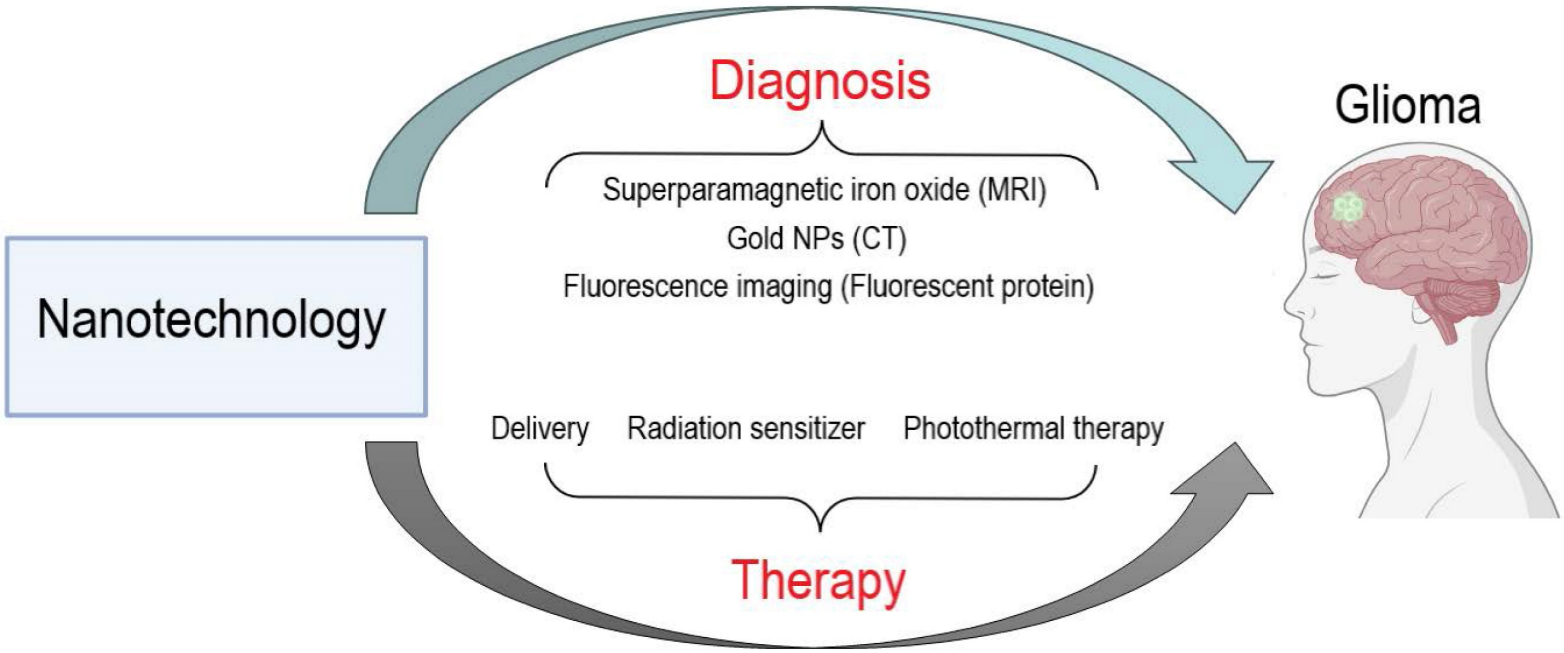
Potential of nanotechnology in the diagnosis and treatment of glioma.

This review systematically discusses the ongoing challenges in glioma treatment and summarizes the potential of nanotechnology-based glioma diagnosis and treatment **(Figure 1)**. This review concludes with an outlook on the future direction of nanotechnology use in glioma therapy to provide a theoretical basis and new insights for glioma research.

## Current Status of Glioma Treatment

Currently, the traditional treatments for gliomas include surgical excision, radiotherapy, total body treatments, localized treatment, and supportive therapy **(Table 1)**. The standardized treatment regimens used in recent decades for glioma treatment include maximum safe range surgical resection, radiation therapy (2 Gray/day, five days/week for six weeks) concurrently with daily temozolomide (TMZ) at 75 mg/m2, and six subsequent cycles of TMZ (150-200 mg/m2) 19. However, the prognosis for patients is still not ideal, as the median overall survival of GBM patients is only 14.6 months, and the 5-year survival rate is only 4-5% 20. There are several reasons for this. First, the maximum safe margin of excision affects the efficacy of subsequent radiation and chemotherapy treatments. Additionally, tumor location affects the extent of resection, and subjective judgment by the attending physician during the procedure also affects the extent of resection 21,22. Therefore, preoperative imaging is essential for determining the extent of surgical resection. Second, although TMZ is a first-line therapeutic agent for glioma, patients are prone to drug resistance, and the drugs have limited effectiveness with long-term use 23. Glioma patients respond differently to TMZ depending on the methylation of the O6-methylguanine-DNA methyltransferase (MGMT) promoter 24. Long-term alkylating agent therapy increases the risk of myelodysplasia and increases the cost of care. Moreover, DNA damage induced by radiotherapy under hypoxic conditions can be repaired by intracellular DNA repair enzymes, thereby increasing cellular tolerance to radiotherapy 25.

In summary, standardized treatments have inherent limitations. The addition of bevacizumab, which targets vascular growth factors, to radiotherapy/TMZ has been shown to improve progression-free survival (PFS), improve quality of life and reduce steroid requirements. However, it does not significantly improve overall patient survival 26,27. Treatment with electric fields (TTFs) in combination with TMZ as adjuvant therapy has been approved, but its cost, treatment compliance, and skin toxicity limit its use 28,29. The slow development of targeted therapies and immunotherapies for glioma is related to its high heterogeneity and mechanisms governing immunosuppression and acquired resistance. In contrast, with the rapid advancement of nanotechnology, nanomaterials with unique physical, chemical, and biological properties are gaining prominence in biomedicine, providing new and exciting therapeutic strategies for glioma treatment.

## Challenges in Glioma Treatment

**Figure 2:**
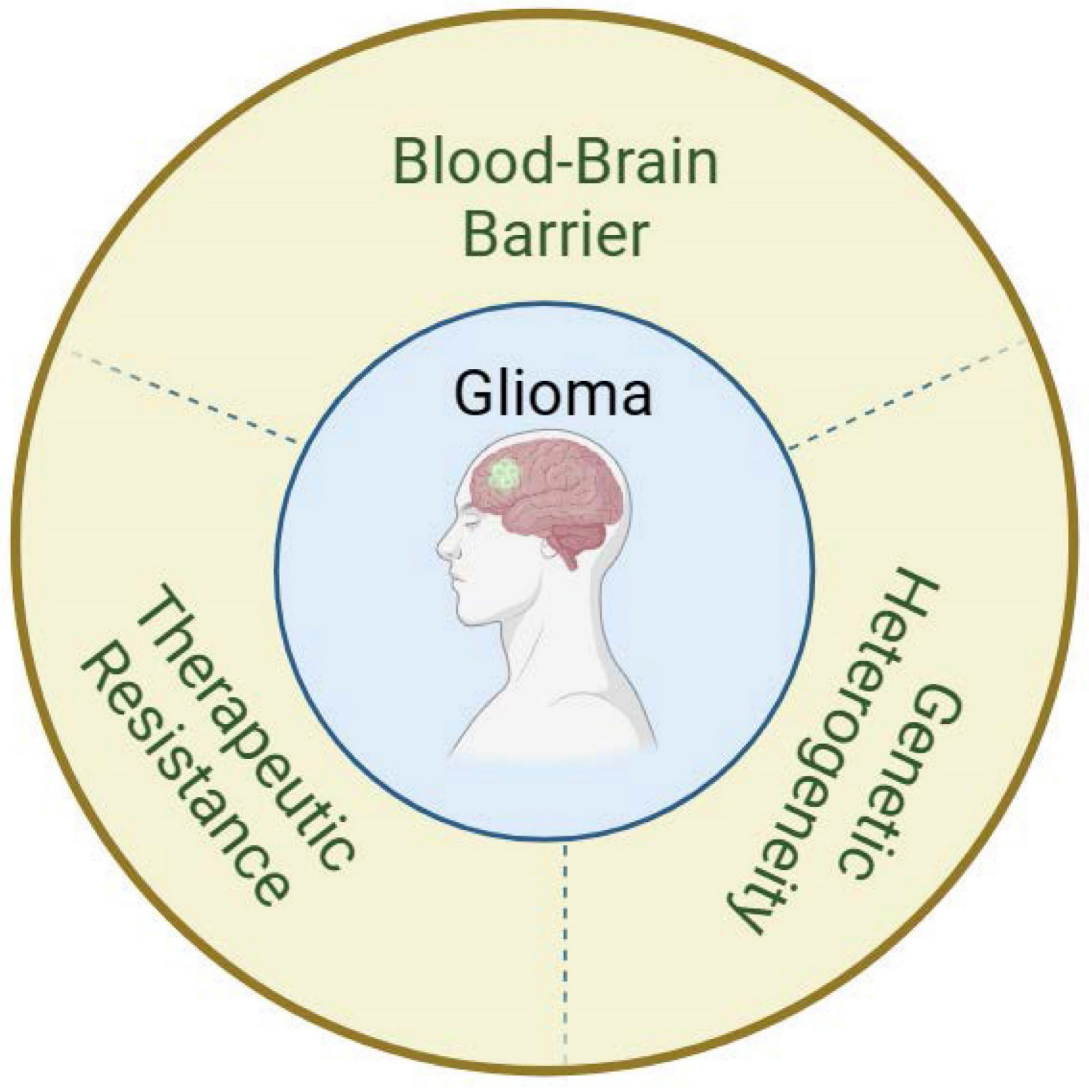
Major challenges in the treatment of glioma.

To date, effective therapies that improve the long-term survival of glioma patients are lacking. This paragraph summarizes the major challenges currently facing glioma treatment, including the blood‒brain barrier, therapeutic resistance, and genetic heterogeneity** (Figure 2)**.

### BBB

The BBB is the barrier between plasma and brain cells formed by capillary walls and glia of the central nervous system. It is essential for maintaining the stability of the brain's internal environment 30. First, the BBB allows small molecules necessary to sustain neural function, such as glucose, amino acids and organic ions, to enter the brain 31. On the other hand, the BBB effectively prevents toxic substances in the peripheral blood from entering the brain, protecting brain tissue from damage 32. However, the BBB likewise precludes the transit of most drugs into the brain, making it difficult for chemotherapeutic agents to reach therapeutically effective concentrations in brain tissue. Increasing the dose can cause severe toxic side effects, which severely limits the treatment of CNS disorders 33.

The integrity of the BBB may be affected by gliomas, resulting in enhanced permeability and substance transport capacity 34. However, such changes exist only in localized regions affected by gliomas and result in a relatively low degree of increase in BBB permeability, which is inadequate for many therapeutic agents to enter the brain. Although research targeting BBB permeability has undergone rapid development, including modes of drug delivery that increase permeability and bypass the BBB, progress in conducting clinical trials has been very limited. To date, nanoparticle delivery systems remain the most promising option for overcoming the challenges posed by the BBB. For example, nanoparticles modified by hydrophilic moieties or BBB endothelial cell surface-specific receptors exhibit strong trans-BBB capabilities and are considered to be the most desirable drug delivery system for glioma therapy 35.

### Therapeutic Resistance

Numerous anticancer drugs have been used to treat gliomas but the prognosis of glioma patients has remained poor, which may be attributed to unique resistance mechanisms. Acquired chemoresistance in tumor cells is caused by drug-induced genetic and epigenetic changes 36. Studies have shown that tumor cells with stem cell characteristics are directly involved in brain tumor recurrence and drug resistance 37. During glioma treatment, TMZ kills drug-sensitive tumor cells. At the same time, drug-resistant tumor stem cells may proliferate in large numbers and become the dominant cell population. MGMT directly repairs TMZ-induced damage to tumor cell DNA, a significant cause of glioma drug resistance 38. Clinical studies have shown that patients with methylated MGMT gene promoters are more sensitive to TMZ treatment 39. Methylation of the MGMT gene promoter has become a predictive marker for the effectiveness of treatment with alkylating agents such as TMZ. DNA base excision repair is primarily involved in non-large-scale DNA damage repair and is an alternative pathway for glioma drug resistance 40. When DNA is damaged by TMZ, poly (ADP-ribose) polymerase-1 (PARP-1) binds to gaps in DNA single- or double-strand breaks and catalyzes the cleavage of β-nicotinamide adenine dinucleotide to generate nicotinamide and ADP-ribose 40.

Nanotechnology-based approaches have made new advances in overcoming these resistance mechanisms in the last decade. For example, nanotechnology can improve drug delivery and cellular uptake and enhance glioma sensitivity to chemotherapeutic agents 41. In addition, nanotechnology enables delivery of siRNAs and peptides, which can significantly inhibit glioma resistance to TMZ or other chemotherapy drugs 41. A recent report claimed that combining multimodal neuro- and nanotechnology-enabled precision immunotherapies with existing systemic immunotherapies could overcome therapeutic resistance may ultimately provide a major breakthrough against GBM 42. From this perspective, the development of nanotechnology in combination with existing therapies might become an emerging treatment modality for gliomas.

### Genetic Heterogeneity

Glioma exhibits substantial genetic heterogeneity, which is thought to be a key factor driving therapeutic resistance and tumor recurrence. Gliomas may have different mutations or expression levels of genes, including those related to oncogenic signaling axes, metabolism, and immune response 43-45. The generation of phenotypic heterogeneity in gliomas differs from the hierarchical differentiation process of normal stem cells. Neural stem cells irreversibly give rise to stereotypical progenitors and differentiated cells during unidirectional stratification. However, there is little difference between glioma stem cells and stem cells that give rise to diverse cell populations; these cells are highly plastic 46. Glioma cells have multiple states, including neural progenitor cell (NPC)-like, oligodendrocyte progenitor cell (OPC)-like, astrocyte (Astro)-like, and mesenchymal (Mes)-like differentiation-like states 47,48. The genetic heterogeneity of gliomas allows gliomas to acquire different abilities in hypoxic environments, immune escape mechanisms, and chemotherapeutic drug sensitivities 49-51. Therefore, recognizing the distinct genetic makeup of each patient's tumor is critical for developing strategies for personalized medicine. With the development of molecular diagnostics, the classification of various glioma subtypes has been improved, which has potential implications for the development of personalized nanotechnology-based therapies. For example, designing NPs that specifically bind to surface receptors expressed on glioma cells could be effective in promoting cellular uptake and improving therapeutic efficacy 52. In addition, cellular and molecular studies focused on understanding the GBM microenvironment, especially in the aggressive peripheral regions, provide a wealth of information for the design of specific nanotherapeutics. Several potentially effective nanotherapeutics are currently in clinical trials. For example, a Phase II clinical trial is currently studying the effects of a combination of TMZ and intravenous targeted p53 gene therapy (a cationic liposome, SGT-53) (NCT02340156). Although it is hoped that these trials will lead to promising new treatments for glioma, more research is needed to determine ultimate efficacy, feasibility and safety.

## Nanotechnology for Glioblastoma Diagnosis

Accurate diagnosis is the most crucial step in glioma treatment. Since gliomas grow invasively in the brain, it is challenging to accurately locate the exact tumor boundary using clinical imaging techniques. The failure of surgery to completely remove the tumor is an essential reason for the high recurrence rate and high mortality rate of GBM 53. Therefore, there is an urgent need for more advanced GBM diagnosis and treatment methods, and nanotechnology has shown great potential in glioma prediction, diagnosis, imaging and therapy.

To achieve a more accurate diagnosis of gliomas, the small particle size, photosensitivity, and magnetic properties of nanomaterials are considerable advantages. In addition, nanomaterials can carry many radioisotopes, improving the specificity and sensitivity of imaging, and glioma visualization contributes significantly to the accuracy of glioma diagnosis. Magnetic resonance imaging (MRI), computed tomography (CT), and optical imaging are the most commonly used methods for diagnostic glioma imaging. In recent years, many studies have investigated the combined use of nanotechnology and imaging techniques for diagnosing gliomas.

### Nanotechnology for MRI

MRI is a relatively new medical imaging technique widely used to provide physiological and anatomical information with high spatial and soft-tissue resolution by recognizing differences in the relaxation of hydrogen atoms abundant in flowing water in living organisms. Currently, MRI is the predominant test for the diagnosis of gliomas. However, as clinical needs increase, the background noise, sensitivity and overall resolution of gadolinium-based contrast agents are no longer sufficient for glioma diagnosis. Highly efficient nanoscale contrast agents with high magnetic relaxation and specificity have received much attention and may improve the sensitivity of MRI and better differentiate between diseased and healthy tissues.

Some essential properties of nanomaterials, such as BBB permeability, biocompatibility, specific targeting and low toxicity, make them ideal candidates for MRI of intracranial lesions. Metal-based NPs, such as gadolinium, manganese, and iron, have advanced the study of MRI contrast agents due to their paramagnetic and superparamagnetic properties. Tan et al. developed a superparamagnetic iron oxide (SPIO) nanoprobe for enhanced t2-weighted MRI, and covalent modification with interleukin-6 receptor-targeting peptide (I6P7) allowed the particles to pass through the blood‒brain barrier and to better identify low-grade gliomas 54. There is a need to thoroughly investigate the potential of magnetic nanomaterials for MRI-guided drug delivery in the diagnosis and treatment of malignant brain tumors in the future. In addition, various fluorinated contrast agents are considered promising imaging techniques for cancer diagnosis due to their excellent soft tissue resolution, deep tissue penetration, and virtually no endogenous interference. In recent years, researchers have designed a variety of fluorine MRI (19F MRI) contrast agents and demonstrated satisfactory relaxation efficiencies by further integrating various nanomaterials 55. To avoid the limitations of a single imaging method, the development of multimodal imaging methods in which nanoparticles are combined with multiple imaging methods has recently become favored. For example, MnO NPs with excitation-dependent fluorescence have been synthesized by the thermal decomposition of manganese-based compounds, and this method of combining MRI and fluorescence imaging has shown equally impressive advantages 56. Although there is still a long way to go before these nanomaterials can be used as alternatives to gadolinium-based contrast agents or other commercial materials in clinical applications, these studies support the use of nanotechnology for the clinical diagnosis of gliomas.

### Computerized Tomography Nanoprobe

Electron computed tomography (ECT), single electron emission computed tomography (SPECT) and positron emission tomography (PET) are all commonly used imaging methods for the clinical diagnosis of gliomas. X-rays often require the addition of contrast material to better visualize the differences between glioma and normal brain tissue. The most commonly used iodine agent has a short imaging time, low spatial resolution and poor specificity. Compared with iodine agents, gold nanoparticles have been shown to provide sharper and more stable images 57. Gold nanoparticles are also relatively easy to modify, allowing for better penetration of the BBB and specific targeting of gliomas. In addition, nanomaterials can bind to radioisotopes and target molecules, increasing the amount of contrast agent available to the tumor lesion. Injection of radioisotope-labeled nanomaterials into gliomas has been reported in both imaging and treatment of tumors 58. In conclusion, nanotechnology-based radiation modalities may be a practical breakthrough for providing more detailed and precise anatomical information.

### Fluorescent Nanoprobe

In a study combining chronic brain opening and two-photon microscopy, Zhang et al. established a mouse glioma model *in situ* in a pure syncytiotrophoblast. They investigated the dynamics of nanoparticles during the long-term growth of gliomas. The results showed that silicon nanoparticles (SNPs-PEG-RGD-FITC) bound by intravenous injection of integrin αvβ3 had high penetration and retention in solid gliomas, revealing the dynamic real-time observation of nanoparticles in a mouse glioma model and laying a technical foundation for exploring the targeting and infiltration of nanomaterials in gliomas 59. A recent study reported a glioma targeting and redox activatable theranostic nanoprobe (Co-NP-RGD1/1) for magnetic resonance (MR) and fluorescence (FL) bimodal imaging-guided on-demand synergistic chemotherapy/photodynamic therapy (Chemo-PDT) of orthotopic gliomas 60. In an *in vitro* model, Co-NP-RGD administered intravenously could be delivered to *in situ* glioma cells across the BBB in the presence of cRGD-targeting groups on the surface, thus generating an enhanced MRI contrast signal for the localization of *in situ* gliomas in the brain 60. Sheng et al. developed a new novel multimodal nanoprobe of NIR-II fluorescent molecules with aggregation-induced emission (AIE) properties based on the principle of NIR-II fluorescence imaging 61. They achieved high-resolution, high signal-to-noise ratio dual-modal molecular imaging of gliomas via NIR two-region fluorescence and NIR one-region photoacoustic imaging in a mouse glioma model. In addition, other domestic and international studies have made considerable research progress in multimodal nanoprobe guidance for determining the intraoperative boundaries of gliomas, which provides a new guidance technique for the resection of gliomas 62.

### Nanotechnology for Molecular Diagnostics

The rapid development of whole genome sequencing (WGS) has accelerated the development of precision medicine by providing unprecedented information on genotypes and phenotypes of diseases. The search for disease-related biomarkers has become a hot topic in recent years, especially in tumor research. Molecular biomarkers (e.g., genes, RNAs, and proteins) have been widely used for disease prediction, diagnosis, and prognostic assessment 63. In recent years, molecular pathology of gliomas has made significant progress. In 2016, WHO has included molecular pathology into the pathological diagnostic system of gliomas, which provides a differential basis for tumors that are difficult to be clearly diagnosed by histology, which is conducive to a better judgment of the clinical prognosis of patients 64. For example, IDH mutations, combined chromosome 1p/19q deletions and MGMT promoter methylation are newly identified molecular variants that have the potential to become new targets for future glioma therapy 64. Surface plasmon resonance (SPR) sensing is a novel analytical technique developed based on optical principles and has been shown to have great potential for studying molecular interactions. Recent studies have found that SPR measurement of patient miRNA-182 levels in combination with nanotechnology is expected to aid in the diagnosis of gliomas 65. In addition, CRISPR/Cas13a RNA-editing system is a novel molecular diagnostic tool with the advantages of rapidity, high sensitivity, and programmability. Wu et al. reported the potential of an *in vivo* imaging method based on CRISPR/Cas13a RNA-editing system in the diagnosis of glioma 66.

In summary, the successful application of nanotechnology in glioma imaging can improve the accuracy of glioma diagnosis and provide a new direction and idea for the accurate diagnosis of glioma in the future. However, the application of nanotechnology to the diagnosis of glioma still has limitations and challenges to be solved. The potential limitations of current nanotechnology mainly include: (i) lack of target specificity, (ii) adhesive interactions with non-target structures, and (iii) biosafety. For example, non-specific adsorption of metal nanoparticles limits their further application in SPR 67. Therefore, in response to the limitations faced by nanotechnology, we expect the next generation of nanomedicine to continue to develop new technologies to overcome these challenges.

## Nanomaterials for Glioblastoma Treatment

### Nanodrug Delivery Across the BBB

Chemotherapy-based nanocarrier technology has become an important research direction to improve the transport of chemotherapeutic drugs across the BBB and increase the efficacy of chemotherapy in GBM treatment **(Table 2)**. Lahann et al. constructed novel synthetic protein nanoparticles (SPNPs) using polymerized human serum albumin (HSA) and the cell-penetrating peptide iRGD 68. The results of mouse experiments showed that synthetic protein nanoparticles (SPNPs) carrying signal transducer and activation of transcription 3 factor (STAT3i) successfully crossed the blood‒brain barrier to reach tumors and enhanced the effect of standardized treatment of GBM after intravenous injection 68. This is the first study to demonstrate that systemic or intravenous delivery of therapeutic agents can cross the blood-brain barrier to reach brain tumors, providing new hope for treating GBM.

Liposomes are considered to be self-assembling colloidal nanocarriers capable of targeted drug delivery to glioma tissues and can be used as carriers for glioma chemotherapeutic agents. For example, human ferritin (HFn) can efficiently cross the BBB by transcytosis through binding to the transferrin receptor (TfR1) on the BBB 69. Different glioma tumor-targeting peptides (including RGE, Pep-1 and CGKRK) were genetically engineered at the N-terminal end of the protein subunit of the heavy chain HFn to confer tumor-targeting and tissue-permeable properties to HFn nanocarriers, while the peptide-modified protein subunits could still self-assemble to form nanocarriers. Modification with the permeable peptide RGE significantly improved the ability of HFn nanocarriers to penetrate deep into tumors and target tumor cells without affecting the ability of HFn nanoparticles to efficiently cross the BBB, proving that RGE-HFn nanocarriers can be used as tumor-targeting and tissue-permeable nanocarriers. HFn nanocarriers can be used as an ideal drug delivery vehicle for targeting gliomas *in situ* 70. Finally, RGE-HFn nanoparticles encapsulating SR717 also showed excellent antiglioma efficacy in animal models, significantly prolonging the survival of the animals. However, its efficacy in humans needs to be further confirmed. Mesenchymal-epithelial transition is associated with poor prognosis, aberrant aggressiveness, and tumor resistance in patients with gliomas and is a possible therapeutic target for gliomas 71.

Polymeric nanoparticles are considered promising materials for drug delivery. Wu et al. coupled epithelial transition factor (MET)-targeted cMBP peptides to G4 dendrimers to form new nanoinhibitors 72. These subsequent experiments showed that this nanoinhibitor effectively reduced glioma cell proliferation and invasion in an *in vitro* model by blocking MET signaling at significantly reduced levels of phosphorylated MET (p-MET) and its downstream signaling proteins, such as p-AKT and p-ERK1/2. Sun et al. developed a novel targeted antisense miRNA-21 oligonucleotide (ATMO-21) delivery system for GBM therapy: bradykinin ligand agonist-modified spermine-modified acetylated dextran nanoparticles (B1L@SpAcDex-ATMO-21 NPs). It can improve the therapeutic effect of drugs on GBM by activating G protein-coupled receptors expressed in tumor blood vessels and tumor cells, resulting in increased BBB permeability and drug transport and accumulation at the tumor site 73.

X-ray-induced photodynamic therapy (XPDT) is highly advantageous for treating deep-seated tumors 74. Based on this idea, Li et al. developed a new CaF2 nanocrystal oscillator with a dendritic polymer as a framework to build a nanoplatform with a dual-core satellite structure that is capable of programmatically and spatially separating the dual loading of therapeutic agents, permitting the combination of XPDT and antiangiogenic therapies, thus generating a restorative system capable of synergistically combating tumors 75. Chai et al. prepared a bionic nanodelivery system composed of high-drug-loading-capacity erythrocyte membrane-coated drug nanocrystals, namely, membrane-coated drug NCs (RBC-NCs), by an antibiotic-biotin interaction insertion method 76. The nanodelivery system can increase target recognition, increase drug accumulation at the tumor site and improve the efficacy of chemotherapeutic drugs against subcutaneous graft tumors and gliomas *in situ*. C-X-C motif chemokine ligand-12/C-X-C motif chemokine receptor-4 (CXCR4-CXCL12) is associated with progression in GBM 77. Although AMD3100 can be used as a GBM therapeutic target and CXCR4 antagonist, achieving successful transport through the BBB and high drug concentrations at the tumor site is difficult. Therefore, Alghamri et al. prepared synthetic protein nanoparticles (SPNPs) coated with transcystic peptides (iRGDs) (AMD3100-SPNPs) and experimentally demonstrated that AMD3100-SPNPs could inhibit GBM cell proliferation and infiltration by blocking CXCL12/CXCR4 signaling 78. The existence of the blood‒brain barrier prevents most drugs from entering the brain and exerting their therapeutic effects, which is a common challenge in treating almost all central nervous system diseases. In contrast, nanomedicines improve therapeutic efficacy by enabling effective drug concentrations in the tumor region of the brain through different means, including BBB permeability reduction or tumor-targeted trans-BBB transport nanodelivery systems. Therefore, the use of a nanodrug delivery platform is a promising direction for GBM therapy.

### Nanomaterials Improve GBM Sensitivity to Treatment

While the first-line chemotherapeutic agent for glioma, TMZ, improves survival in patients with glioma by methylating DNA and inducing cytotoxicity, the BBB and acquisition of drug resistance impact treatment outcomes 79-81. Chemotherapy or postoperative adjuvant chemotherapy can improve patient survival to a certain extent. Scholtyssek et al. conducted a meta-analysis and reported that the 1-year survival of patients treated with a combination of radiotherapy and chemotherapy was greater than that of patients treated with radiotherapy alone and that chemotherapy can indeed prolong patient survival 82. Therefore, improving the sensitivity of gliomas to TMZ is a viable future research direction.

Several recent studies on nanomedicine delivery platforms have shown possible avenues for improving resistance to TMZ. Zhang's team developed a new nanoformulation called RPDGs targeting GBM cells, which utilizes the process of reducing Pt (IV) to Pt (II) to deplete glutathione (GSH) to further increase the level of reactive oxygen species (ROS) in tumor cells, promoting apoptosis and iron death. The anti-GBM effect of RPDGs was confirmed in both *in vivo* and *in vitro* models 83. Platinum compounds are the mainstays of combination drugs used for precision therapy and immunotherapy and have potent anticancer effects. The mechanism of action is mainly the formation of intra- and interstrand cross-links with DNA, which are difficult to repair by MGMT enzymes and are not cross-resistant to DNA-alkylating agents 84. Recently, in Wang et al.'s study, platinum was synthesized by designing and synthesizing the reduction-responsive polymer poly(1,2,4,5-cyclohexane tetracarboxylic acid dianhydride-co-hydroxyethyl disulfide)-polyethylene glycol (poly(CHTA-co-HD)-PEG), which encapsulates a tetravalent platinum prodrug of oxaliplatin (OxaPt(IV)) as well as the DNA-embedding agent 56MESS to form platinum drugs, creating a variety of platinum-containing NPs 85. Platinum-containing nanopharmaceuticals can reverse the drug resistance to TMZ at the cellular level, and in an animal *in situ* tumor model, combined with convection-enhanced drug delivery technology, they can increase the survival period of animals and reverse the resistance of GBM to TMZ 85.

Sonodynamic therapy (SDT) is an emerging treatment method in which ultrasound waves can be precisely applied to the deep tumor area, thus effectively stimulating the acoustic sensitizers enriched in the tumor tissue to generate reactive oxygen species (ROS), resulting in deep and noninvasive treatment while causing minor damage to normal tissues; this method is now used to treat a wide range of diseases 86. Previous studies have demonstrated that it is possible to abrogate the downregulation of chemoresistance-related markers (e.g., HSV-1, p53, and P-gp), reduce drug resistance and improve drug sensitivity by drug and chemo-photothermal combination therapy, which can significantly enhance anticancer efficacy 87. Chen et al. combined polyglutamic acid (PGA) with a chemotherapeutic drug-cum-acoustic sensitizer (adriamycin, DOX), wrapped them in a brain glioma cell membrane, and prepared bionic nanoacoustic sensitizers (MDNPs) with the functions of homologous targeting of brain gliomas and ultrasound-triggered acoustic chemotherapy 88. The MDNPs had suitable homologous targeting ability and effectively penetrated the BBB when combined with ultrasound. Moreover, they reached the intracranial tumor site, induced tumor cell apoptosis, downregulated drug resistance-related factors, disrupted chemotherapy resistance, increased sensitivity to chemotherapy, and significantly prolonged the survival time of mice 88.

In the treatment of glioma, in addition to killing tumor cells, radiotherapy (RT) can have toxic effects on surrounding normal brain cells, thus affecting the efficacy of RT 89. Tumor hypoxia is one of the most important reasons for resistance to radiotherapy 90,91. Therapeutic radionuclides have the potential to increase the therapeutic efficacy of glioma radiation. The liposome-encapsulated radionuclide rhenium has the potential to promote the retention of radioisotopes in tissues. Nanoliposomes help radionuclide rhenium deliver radiation to tumor cells through convection-enhanced delivery 92. The FDA approved the Rhenium-186-NanoLiposome (RNL) for the treatment of patients with recurrent glioblastoma. RNL administers high doses of radiation directly to brain tumors at concentrations up to 25 times that of external beam radiation while being safe, effective and convenient. The results of the phase 1 ReSPECT glioma trial showed that RNL at doses exceeding 100 Gy improved overall survival in patients with recurrent gliomas 93. In conclusion, existing nanotechnology can enhance the sensitivity of glioma to radiotherapy in various ways and open up new avenues for treating glioma.

### Novel Nanomedicine Applications in GBM Therapy

**Figure 3:**
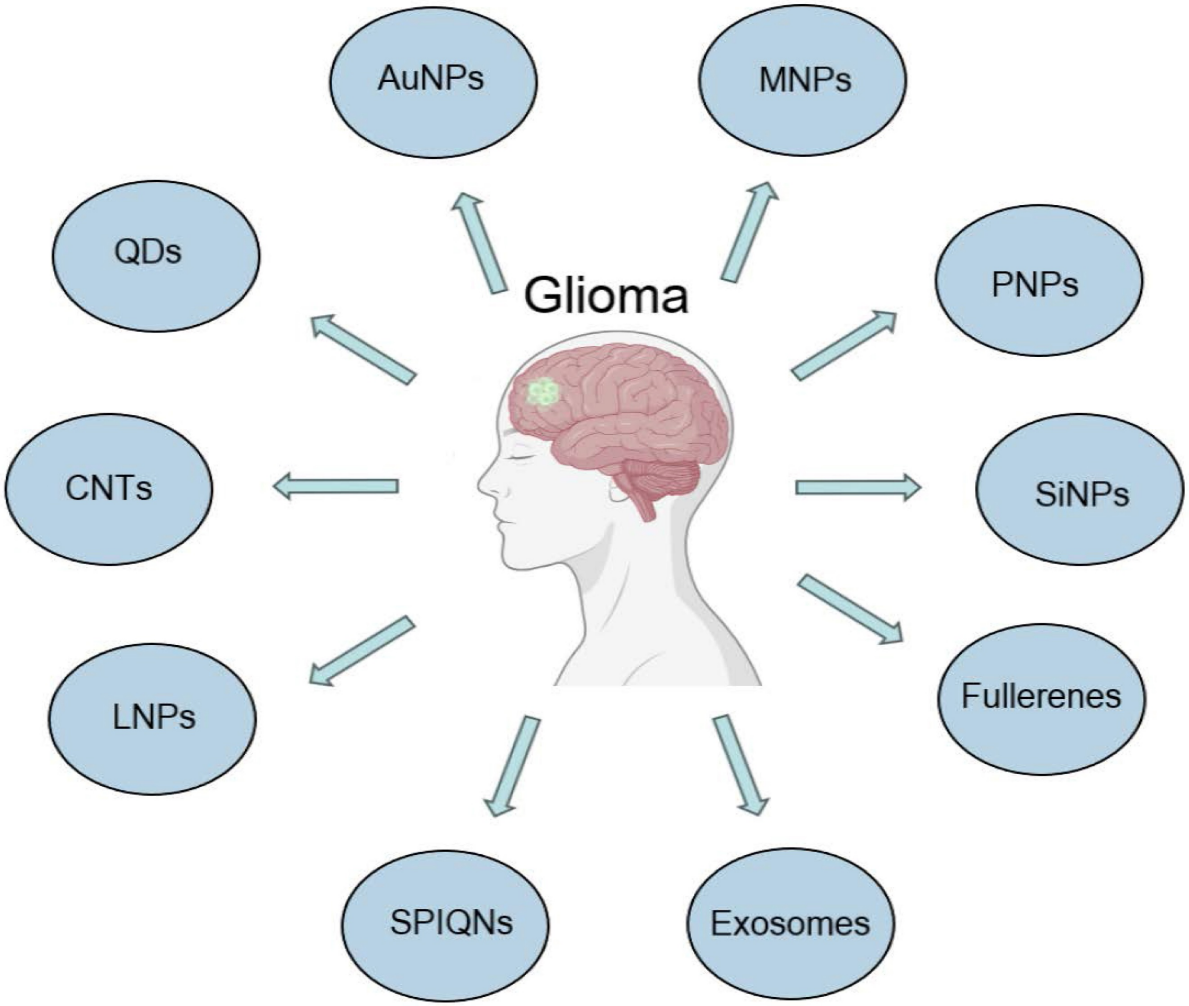
Recently developed nanomaterials in the diagnosis and treatment of glioblastoma.

As research on nanotechnology continues to progress and intersect with that in the medical field, many new nanomaterials continue to be synthesized and investigated for applications in glioma therapy **(Figure 3)**. Exosomes are cell-derived small extracellular vesicles (sEVs) approximately 100 nanometers in size that transport specific substances to target cells 94. Exosomes, which are enriched in proteins, lipids, DNA, RNA and glucose, regulate the extracellular matrix and signal to other cells, affecting all areas of cell biology 95. Exosomes are able to cross the BBB, which makes them possible therapeutic agents and diagnostic tools for gliomas 96,97. Serum exosomal EGFRvIII mRNA has been studied in patients with gliomas and may provide sufficient diagnostic information 98. Conventional nanoparticles (e.g., liposomes or metal particles) have relatively poor bioactivity, compatibility and tumor selectivity with limited benefits 99.

In contrast, exosomes, as a novel nanomedicine delivery technology with low toxicity, improved biocompatibility, and suitable stability, can be potential carriers and effectively stimulate antiglioma immune responses 99. Zhu et al. designed sEVs with dual-targeting functionalization of angiopep-2 and TAT and applied them to the therapeutic study of glioma 100. Angiopep-2 peptide can specifically target low-density lipoprotein receptor (LRP-1), which is highly expressed on the surface of glioma cells and cerebrovascular endothelial cells 101. TAT enhances sEV BBB permeability and tumor tissue penetration. Furthermore, in a mouse glioma model *in situ*, dual-targeted functionalized sEVs were shown to effectively penetrate the blood‒brain barrier and target glioma lesion areas 100. In conclusion, dual-targeted functionalized sEVs can improve the efficiency of drug treatment for *in situ* gliomas, significantly inhibit glioma growth, and effectively prolong the survival of tumor-bearing mice, which provides encouraging prospects for the treatment of gliomas.

Moreover, bionic nanorobots will also play an essential role in medicine in the future. Deng et al. generated natural killer (NK) cell membranes wrapped with aggregation-induced luminescence (AIE) organic semiconductor skeletal materials with near-infrared two-region fluorescence properties to fabricate NK cell-mimicking AIE nanorobots (NK@AIE dots) and evaluated their efficacy in the diagnosis and treatment of glioma 102. NK@AIEdots cross the BBB by regulating the tight junctions (TJs) between BBB epidermal cells through specific interactions with the cells on the BBB, increasing BBB permeability, and enriching glioma cells by the particular recognition of NK cell membranes and glioma cell membrane surface receptors to achieve high signal-to-noise ratio near-infrared fluorescence imaging of brain tumors. The thermal effect generated by NK@AIEdots under near-infrared light irradiation significantly inhibited the growth of glioma tumors. In addition, a recently reported technique of mechanical nanosurgery using magnetic carbon nanotubes (mCNTs) and precise magnetic field control was developed based on the principle that mCNTs are enriched in GBM, and mechanical stimulation using magnetic field control can destroy the cellular structure and lead to tumor cell death 103.

Recent studies have demonstrated that nanoimmunotherapy, a type of nanoparticle-based tumor immunotherapy, has great potential as a personalized and synergistic treatment regimen due to its unique biological properties that facilitate precise targeting, local drug delivery, and enhanced therapeutic efficacy. Exosome-based nanoimmunotherapy targeting tumor-associated macrophages (TAMs) is a promising immunotherapeutic strategy that uses nanosized exosomes as a delivery system for specific therapeutic agents targeting TAMs to modulate their behavior and inhibit the recruitment or deplete or reprogram them from a tumor-promoting phenotype to a tumor-suppressing phenotype 104.

### Novel Nanomedicine Applications in GBM Therapy advantages and disadvantages of the Nanotechnology on the diagnosis and treatment of gliomas

More and more advances have been made in novel nanotechnology-based diagnostic and therapeutic systems for gliomas. Next there is an urgent need to validate the efficacy of these nanotechnologies in clinical practice. AGuIX NPs in combination with radiotherapy have been found to be clinically beneficial for glioma patients in recent phase Ib/II clinical trials (NCT04881032) 105. However, the only NP available for clinical application to date, ferumoxytol, has caused rare but severe allergic reactions 106. Therefore, the application of nanotechnology to the clinical diagnosis and treatment of gliomas still faces many challenges.

Nanotechnology offers several advantages in the diagnosis and treatment of gliomas, though it also presents some challenges. This technology can integrate multiple functions, including imaging diagnostics, drug delivery, and photothermal therapy, to achieve comprehensive diagnostic and therapeutic solutions. One of its key benefits is improved penetration of the blood-brain barrier (BBB), allowing for targeted drug delivery directly to tumor cells, thereby minimizing the impact on healthy cells. Additionally, encapsulating drugs within nanoparticles can extend their half-life and enhance their solubility and stability. However, there are several significant disadvantages associated with the use of nanotechnology in glioma diagnosis and treatment: 1. Biocompatibility and Toxicity: The long-term biocompatibility and potential toxicity of nanomaterials within the body remain critical concerns. 2. Cost and Complexity: The production of nanotechnology products typically requires sophisticated, high-cost equipment and complex processes, limiting widespread application. 3. Clinical Translation: The process of translating nanotechnology from research to clinical practice is lengthy and intricate. 4. Regulatory Challenges: The approval and oversight of nano-medical products necessitate new evaluation standards and regulatory frameworks. 5. Environmental Impact: The disposal of nanomaterials poses environmental challenges. Despite these challenges, nanotechnology remains a highly promising solution for the diagnosis and treatment of gliomas. As technological advancements continue and further research is conducted, it is anticipated that many of the current limitations will be addressed, thereby enhancing the application of nanotechnology in the medical field.

## Conclusion and Future Expectations

New treatment modalities are urgently needed as the 5-year survival rate of GBM patients is still very low, and improvements in existing treatments are difficult to achieve 107. In recent decades, substantial progress has been made in nanotechnology 108. The use of nanotechnology to develop novel therapeutic strategies for cancer treatment is promising 109. With respect to precision medicine, nanotechnology has excellent potential for GBM diagnosis and treatment. It offers the possibility of individually tailored treatment regimens for each patient, and it provides new options for the treatment of deep-seated tumors as well as for the elimination of residual microtumours or individual tumor cells, which are the leading cause of cancer recurrence 110.

In the future, nanomaterials are expected to be more effective than current cancer treatments, including the surgical removal of tumors, chemotherapy and radiation. Nanocarrier drugs offer many advantages over conventional medicines. They better regulate the pharmacokinetics and drug distribution profiles and can improve drug uptake and intracellular penetration in target cells/tissues 111. However, medical nanocarriers must have suitable biocompatibility and biodegradability and an extended drug circulation half-life 112. They must target specific cells or tissues. In addition, accumulation in off-target organs of the body should be avoided, and clearance strategies should be developed to prevent damage at the time of use. Although there are various methods for generating nanoparticles, ultimately, they must be easy to assemble, cost-effective, homogeneous, strictly conform to clinical trials, highly tissue specific, and safe.

## Author contributions

**Jun Lei**- Acquisition study concept and design- manuscript writing; **Yiyang Huang**, **Yichuan Zhao**, **Zhi Zhou, and Lei Mao**- Study concept and design; **Yanhui Liu**- revision of the manuscript for important intellectual content and study supervision.

## Figures and Tables

**Table 1:** Summary of traditional glioma treatments

Treatment Strategies		Specific measures	Limitations	Improvements
Surgical Excision		Gross Total Resection (GTR). GTR improves survival outcomes and increases survival rates.	Due to the infiltrative growth of GBM in the brain, the tumor boundaries that could not be accurately identified by conventional imaging techniques, the surgery failed to completely resect the infiltrated area of the tumor (47).	Tumor fluorescence with 5-aminolevulinic acid (5-ALA) allows for more complete tumor resection, thereby improving progression-free survival in patients with GBM.
Radiotherapy		Routine postoperative RT was combined with TMZ to deliver 60 Gy of radiotherapy in 2-Gy fractions over a 6-week period (9). Standard first-line chemotherapy consists of TMZ (75 mg/m2 per day) during radiotherapy followed by 6 cycles of temozolomide (150-200 mg/m2 every 28 days for 1-5 days) (17).	At present, other radiation dose options have been explored, but there are no clear benefits. (67)	NA
Total Bodies Treatments	TMZ		Drug toxicities of TMZ include nausea and myelosuppression.	Longer or dose-intensive regimens of temozolomide have not been studied to prove beneficial.
	Procarbazine, Lomustine, Vincristine	Salvage chemotherapy used in combination may have some activity but is limited by greater toxicity.	NA	NA
	Bevacizumab		Bevacizumab improved PFS but did not prolong OS.	Bevacizumab in combination with lomustine improved PFS, but again there was no OS benefit.
Localized Treatment	Tumor-Treating Fields (TTF)	The addition of TTF to temozolomide maintenance chemotherapy improves in terms of progression-free survival and overall survival there (26).	Non-blinding and delays in randomization are the major concerns. Cost, treatment compliance and skin toxicity are other barriers that limit the adoption of this treatment modality.	NA
Supportive Therapy	Antiepileptic Treatment	Most patients with GBM require long-term antiepileptic therapy, and the principle of treatment should be seizure control at low doses to avoid side effects and minimize drug-drug interactions.	Levetiracetam has been most extensively studied in GBM patients and is more an'quan relative to other drugs (20,21).	NA
	Corticosteroids	Corticosteroids are commonly used to reduce peritumoral vasogenic edema; side effects limit long-term corticosteroid use	NA	

**Table 2:** Summary of nanotechnology-based drug delivery across the BBB

Nanomedicine	Components	Mechanism of action	Outcomes
**STAT3i SPNPs**	Synthetic protein nanoparticle (SPNP) particles of polymerized human serum albumin (HSA) equipped with the cell-penetrating peptide iRGD, which contains siRNAs targeting signal transducer and activator of transcription 3 factor (STAT3i) (57).	STAT3 is a critical factor associated with GBM progression, and free siRNA provided a slight benefit with prolonged median survival when combined with radiotherapy (57).	STAT3i SPNPs combined with radiotherapy induces dendritic cell (DC) activation by enhancing the expression of MHC II, which is involved in antigen presentation, and induces an immune response to kill the tumor (57).
**SR717@RGE-HFnNP**	Heavy chain ferritin (HFn) binds tumor-targeting peptides including RGE, Pep-1 and CGKRK to form glioma-targeting RGE-HFn nanocarriers. Then SR717@RGE-HFnNP was formed after the STING agonist SR717 was encapsulated within the RGE-HFn nanocarriers (58).	STING is involved in a variety of diseases. Activation of STING induces an effective immune response against pathogenic infections and cancers, while aberrant activation of STING triggers autoimmune and inflammatory diseases (58).	The biomimetic nanocarrier successfully crossed the BBB and delivered the STING agonist to the glioma lesions in the brain, which effectively activated the STING pathway and triggered the immunomodulatory effect, thus effectively inhibiting the growth of glioma and improving the survival rate of glioma-loaded mice (58).
**Den-cMBP5 and Den-cMBP10(29)**	cMBP is a MET receptor-specific targeting peptide. cMBP peptides were coupled to G4 dendrimers and Den-cMBP5 and Den-cMBP10 were prepared (61).	cMBP is a MET receptor-specific targeting peptide that competes with HGF for binding to the MET site, which prevents the dimerization of the MET receptor and inhibits the activation of its downstream signaling pathway. However, the application of the peptide in targeted therapy is limited by its short cycle time and easy degradation.	The inhibitor exhibited extremely high binding affinity for MET and effectively inhibited glioma growth by blocking MET downstream signaling.
**ATMO-21@SpAcDex NPs(30)**	Antisense miRNA-21 oligonucleotide (ATMO-21) achieves high loading in bradykinin ligand agonist-modified spermine-modified acetylated dextran nanoparticles (SpAcDex NPs).	SpAcDex NPs inhibit tumor growth by activating G protein-coupled receptors, which temporarily open the BBB and deliver ATMO-21.	An *in vivo in situ* glioma model confirmed that ATMO-21 released from SpAcDex NPs inhibits tumor growth and is an effective therapeutic strategy for GBM.
** RGD-RBC-NCs (33)**	Targeted peptides are modified on the surface of red blood cell (RBC) membranes. As RBC membrane-encapsulated drug nanocrystalline NCs (RBC-NCs), RGD-RBC-NCs were formed after modification by tumor-targeting peptide c (RGDyK).	NA	Active target recognition capability to increase drug accumulation at the tumor site improves the efficacy of chemotherapeutic agents against subcutaneous graft tumors and gliomas *in situ*.
**AMD3100-SPNPs(35)**	SPNPs encapsulate the extracellular transcription peptide iRGD (AMD3100-SPNPs) (67).	CXCR4 antagonist (AMD3100) has made an attractive target for anti-GBM therapy.	Blocking CXCL12/CXCR4 signaling. This inhibits GBM proliferation and reduces the infiltration of CXCR4+ M-MDSCs into the tumor microenvironment.
**Adenosine 2A receptor (A2AR) nano-agonists(36)**		A2AR targets neovascularization in glioma-infiltrated areas, prompting vascular endothelial cell cytoskeleton contraction through specific activation of the A2AR signaling pathway, opening tight junctions between endothelial cells, and transiently increasing blood-brain barrier permeability.	Imaging monitors blood-brain barrier permeability and injects the chemotherapeutic drug paclitaxel to increase the drug's efficacy while decreasing its toxicity to normal tissues.
